# Ambient Light Rejection Integrated Circuit for Autonomous Adaptation on a Sub-Retinal Prosthetic System

**DOI:** 10.3390/s21165638

**Published:** 2021-08-21

**Authors:** Hosung Kang, Hojong Choi, Jungsuk Kim

**Affiliations:** 1 Department of Medical Science, Korea University, Seoul 02841, Korea; 2017010569@korea.ac.kr; 2 Department of Medical IT Convergence Engineering, Kumoh National Institute of Technology, 350-27 Gumi-Daero, Gumi 39253, Korea; 3 Department of Biomedical Engineering, Gachon Univesity, Hambakmoe-ro 191, Incheon 21936, Korea

**Keywords:** retina implant, active pixel sensor, image sensor, ambient light

## Abstract

This paper introduces an ambient light rejection (ALR) circuit for the autonomous adaptation of a subretinal implant system. The sub-retinal implants, located beneath a bipolar cell layer, are known to have a significant advantage in spatial resolution by integrating more than a thousand pixels, compared to epi-retinal implants. However, challenges remain regarding current dispersion in high-density retinal implants, and ambient light induces pixel saturation. Thus, the technical issues of ambient light associated with a conventional image processing technique, which lead to high power consumption and area occupation, are still unresolved. Thus, it is necessary to develop a novel image-processing unit to handle ambient light, considering constraints related to power and area. In this paper, we present an ALR circuit as an image-processing unit for sub-retinal implants. We first introduced an ALR algorithm to reduce the ambient light in conventional retinal implants; next, we implemented the ALR algorithm as an application-specific integrated chip (ASIC). The ALR circuit was fabricated using a standard 0.35-μm CMOS process along with an image-sensor-based stimulator, a sensor pixel, and digital blocks. As experimental results, the ALR circuit occupies an area of 190 µm^2^, consumes a power of 3.2 mW and shows a maximum response time of 1.6 s at a light intensity of 20,000 lux. The proposed ALR circuit also has a pixel loss rate of 0.3%. The experimental results show that the ALR circuit leads to a sensor pixel (SP) being autonomously adjusted, depending on the light intensity.

## 1. Introduction

Retinal implants have great promise in restoring vision for the blind, who suffer from retinal diseases such as retinitis pigmentosa and age-related macular degeneration [1,2,3,4]. The fundamental idea for retinal prosthetics is to electrically stimulate impaired retina cells using a microelectrode array and its driving circuitry [5,6,7,8,9]. This retinal prosthesis can be classified into epi-retinal [5,6] and sub-retinal implants [7,8,9], based on the anatomical location. While the epi-retinal implant is placed onto an inner retinal layer, known as the ganglion cells, the subretinal implant is located in the outer retina, called photoreceptor cells. Although the developed implant methods have their advantages and disadvantages, it is widely known that sub-retinal implants can purse for a high resolution of more than 1000 pixels, compared with epi-retinal implants [10,11,12].

It has been reported that high-resolution stimulation pixels can support high visual acuity [12]. According to a clinical trial [11,12], however, the sub-retinal implant with 1500 stimulation pixels shows equal vision restoration compared with the epi-retinal implant with only 60 pixels. This mainly arises from the interface between neighboring pixels during stimulation and a strong ambient light projected onto the subretinal chip. The first interference issue, that results in a current dispersion, becomes more critical when simultaneously stimulating neighboring pixels [13,14]. To suppress the current dispersion, various methods, such as a wall structure between pixels [15] and a sequential stimulation pattern [5,6,16], were applied to the subretinal implant. The second ambient light induces saturation of all stimulation pixels, especially under an outdoor bright environment. As a result, this results in low contrast sensitivity. To solve this issue, an image processing technique was presented in [17,18,19,20]. However, it is challenging to increase the contrast sensitivity from fully statured images. In addition, this processing unit leads to high power consumption and high area occupation, on a limited retinal silicon chip. Another method is to manually adjust the contrast control knob, which is related to the integration time for the photodiode [21,22]. This can cause inconvenience to patients and is tiresome in daily life. Therefore, it is necessary to autonomously cancel out ambient light in the first stage of the retinal implant system.

The ambient-light cancellation system must meet the following three design requirements. Firstly, the system architecture should be realized as small as possible on the limited silicon chip area. An independent imaging processing unit to compensate for the ambient light occupy a big footprint, which can result in the big retinal chip size too. It would be more critical if the number of stimulation pixels increase more than 1000. The large-area retinal chip requires a large incision to insert the chip inside the eyeball. It can cause a side effect, i.e., an infection around the suture site [23]. Secondly, the cancellation circuit must be operated in low-power dissipation. The image processing unit demands to precisely acquire raw data from all the pixels, quickly analyze them, and properly compensate for saturated pixels due to an ambient light. For an image processing, an analog-to-digital data converter is required. In the worst case, one stimulation pixel needs one data converter, which can consume high power in the high-density stimulation retinal chip. Finally, the ambient light must automatically be removed. In reality, an ambient light surrounding patients who have the retinal implant varies with their location. So far, the patients have controlled the knob to avoid a pixel saturation that arises from a bright ambient light. However, many of them feel inconvenienced by the manual compensation method [24]. Accordingly, a low-power and autonomous compensation circuit to get rid of the ambient light must be realized along with high-density stimulation pixels on the retinal chip. 

Motivated by this, we propose a novel ambient light rejection (ALR) circuit to autonomously enhance contrast sensitivity. To cancel out the ambient light, we developed a control circuit to adjust the integration time used for 3-Tr complementary metal-oxide-semiconductor (CMOS) image sensors, where the integration time facilitates sensing the light intensity. The procedure of the control circuit is divided into the detection of pixel saturation and modulation of the integration time. In the case of a bright environment, the 3-tr CMOS image sensor is operated on for a short integration time, while a long integration time is required for a dim environment. This ALR circuit was designed and fabricated using a DongBu Hi-tek 0.35 μm CMOS process and integrated with stimulator pixels, tested on a benchtop environment. 

The remainder of this paper is organized as follows. First, a circuit optimization is operated (as an image-sensor-based stimulator (ISNS) pixel) to obtain the contrast sensitivity depending on the integration time. In addition, an autonomous adaptation optimization takes place to confirm the modulation procedure for the integration time through the ALR circuit. Second, the ALR circuit implementation is presented with the simulated results. Third, the results measured from the ALR circuit with a modulated integration time corresponding to the incident light intensity, are presented. Finally, the design constraints of the proposed ALR circuit are discussed.

## 2. Materials and Methods

### 2.1. ISNS Pixel Design

**Figure 1:**
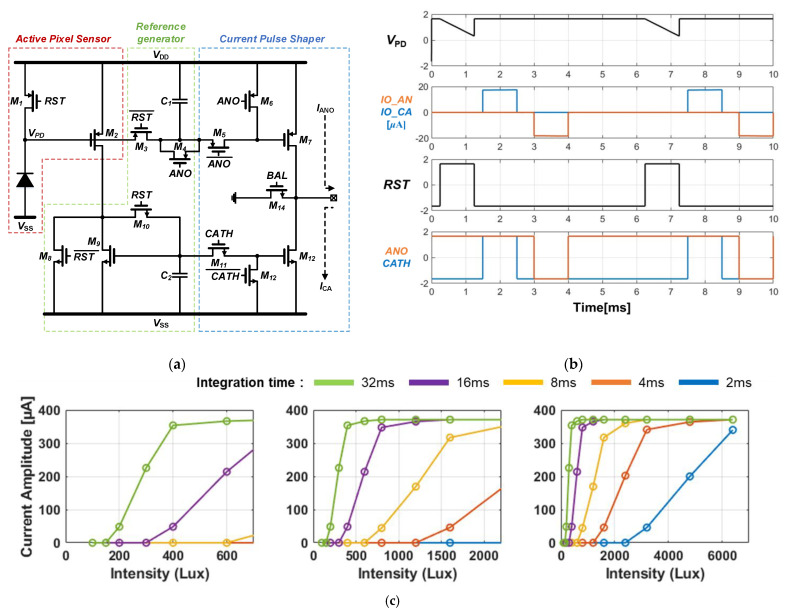
A PBStim pixel circuit with simulation results. (**a**) An image sensor based neural stimulator pixel; (**b**) A simulation results of the ISNS; (**c**) The measured results of ISNS with variable integration time.

Figure 1a,b shows the schematic and simulation of the ISNS pixel scheme. In Figure 1b, the photodiode is replaced with an electrical model with a current source of 6 nA and a parasitic capacitor of 8 pF, which are in parallel. When the reset signal, *RST* is switched to logic “1”, the integration time starts to accumulate a photocurrent until the *RST* is switched off again. During the integration time, the voltage node of *V_PD_* is proportionally decreased, as shown in Equation (1).

(1) $$V_{PD}=V_{DD}-\frac{I_{PD}}{C_{PD}+C_{1}}\cdot T_{int}$$

where *C_PD_* and *I_PD_* indicate the photodiode parasitic capacitor and the photocurrent, respectively. In the reference generator (Figure 1a), *C*_1_ and *C*_2_ capture the final values of *V_PD_* at the end of the integration time and maintain the voltages until the next integration time begins. The restored voltages on *C*_1_ and *C*_2_ generate a cathodic current, *CATH*, and anodic stimulation current, *ANO*, due to the current pulse shaper described in Figure 1a. The *M*_14_ transistor functions as a switch to remove the residual charge after stimulation, which can lead to harmful effects such as electrode erosion [25] and tissue absorption [16,22]. Figure 1c depicts the results of the stimulation current amplitudes corresponding to the variable integration time. 

**Figure 2:**
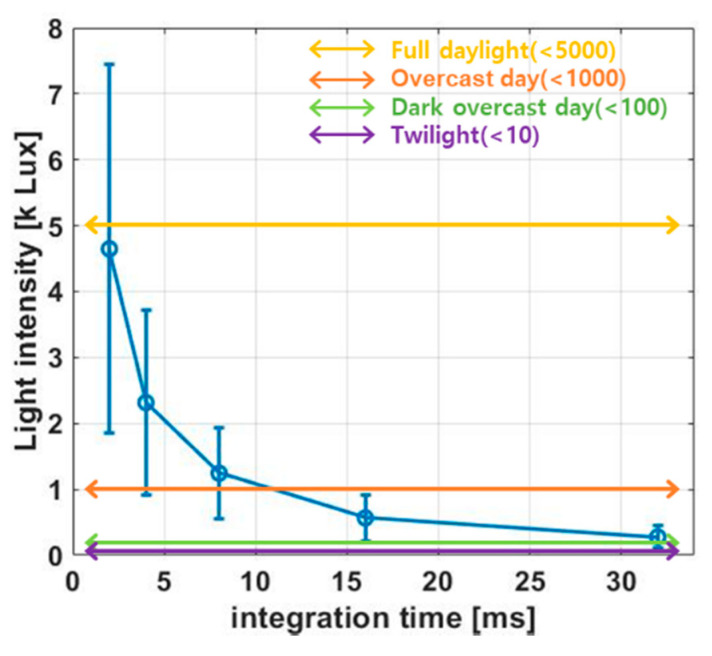
The sensing dynamic range of the ISNS with variable integration time.

Figure 2 shows the sensing dynamic range (SDR) of the ISNS versus the integration time, expressed in Equation (2).

(2) 1Tint·C1+CpK·[2·ImaxμpCox(WL)p−(VDD−VTH.P)]

where *T_int_*, *K*, *I_max_*, and *V_TH.P_* denote the integration time, coefficient for quantum efficiency, maximum current amplitude from the ISNS, and threshold voltage for the *M*_2_ transistor, respectively. Equation (2) shows that the SDR is inversely proportional to the integration time. If an integration time of 16 ms is applied to the ISNS pixel, the SDR would be approximately 300 to 1000 lux. This implies that the sensing dynamic range can be changed by adjusting the integration time. Therefore, it is important to design a circuit that can autonomously vary with the integration time, according to the ambient light intensity. This could prevent the stimulation current saturation. The next section presents a detailed circuit description of the proposed ALR circuit.

### 2.2. Autonomous Adaptation Optimization

**Figure 3:**
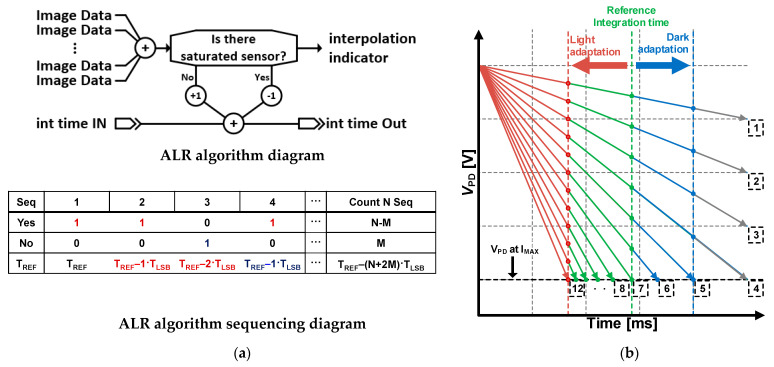
(**a**) An interpolation algorithm and a timing diagram and (**b**) an implementation example.

Figure 3a presents the ALR algorithm for adjusting the length of the integration time. Here, the integration time controlled by the ALR algorithm varies in accordance with the pixel saturation denoted as “yes” or “no.” If there is a saturated sensor in the accumulated image data, the integration time of the next sequence is shorter or longer. *T_REF_* is the reference integration time, and *T_LSB_* is the time variance when changing the least significant bit (LSB) for the integration time control. Therefore, the addition (or subtraction) of *T_LSB_* to (or from) *T_REF_* results in the integration time affecting the indicator. This simple, yet effective algorithm, autonomously provides an adequate integration time for the ISNS pixel. 

Figure 3b shows an example implementation of the ALR algorithm with differential *V_PD_* decrements during the integration period. We employed 12 individual *V_PD_* decrements, referring to incident light, and tagged each *V_PD_* to express light intensity. For instance, the *V_PD_* tagged on 12 is the brightest condition, whereas *V_PD_* tagged on 1 indicates the dimmest condition. To show the integration time variation for both dim and bright conditions, we set the reference integration time at the middle of the x-axis. First, assuming that the retina implant is exposed to bright conditions, the ISNS pixel on tag 12 is saturated because the integration time is initially set as the reference integration time. Second, the ALR algorithm can detect the pixel saturation from tag 12 and then control the integration time. The ALR algorithm continues to reduce the integration time until the indicator for pixel saturation changes to “no.” Finally, when the integration time generated through the ALR algorithm becomes shorter than the reference integration time, saturated pixels with tags 8–12 operate in the SDR again. This procedure is similar to the light adaptation observed in the human eye. In contrast, dark adaptation is conducted where the retina implant operates under dim conditions, which means that the integration time extension is longer than the reference integration time. To model the dark adaptation, we assumed that the retina implant was exposed to the dim conditions, and the brightest light at that time was tag 5. The indicator for pixel saturation turns to “no” when the ISNS pixel is driven on the reference integration time; thus, the ALR algorithm extends the integration time until the indicator for pixel saturation is converted to “yes.” The brightest light is tag 5 because the ISNS pixel on tag 5 is first saturated. Subsequently, the indicator for pixel saturation becomes “yes,” ceasing the integration time. By increasing the integration time, other pixels on tags 1–4 have precise visual information.

**Figure 4:**
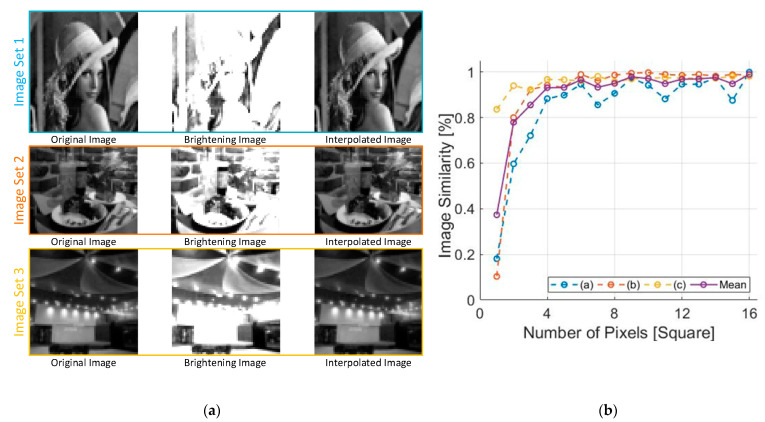
A (**a**) Interpolation image example; (**b**) An image similarity versus number of pixels.

The ALR algorithm shown on Figure 4 is verified with computational simulation. The simulation was performed using MATLAB (MathWorks Inc., Natick, MA, USA). The obtained images had a single dimension of 64 × 64 pixels. We created a brightening image by increasing the brightness of the original image; thus, it partially shows loosened image data caused by pixel saturation. The interpolated images were reconstructed from the brightening images, using the ALR algorithm. These are visually similar to the original images; therefore, we can quantify the similarity between the original and interpolated images. In this study, we prepared three image sets (as shown in Figure 4a) to verify the proposed ALR algorithm on different images. Figure 4b shows the similarity in the number of pixels. Here, the x-axis represents the number of pixels employed in the interpolation, as shown in Figure 3a. As shown in Figure 4b, the image similarity increases up to 100% after 42 pixels. Accordingly, the ALR algorithm achieved the best performance, employing more than 1% of the entire pixel. However, we selected 16 pixels for the ALR algorithm implementation, although employing more pixels ensured high image similarity. This is because the image similarities, calculated on three different images, were over 90% when employing 16 pixels. In addition, the interpolated image in Figure 4a is actually the result of the ALR interpolation performed with 16 employed pixels. Moreover, the increments of the image similarity versus the number of pixels are decreased with an increasing number of pixels. This is related to the power consumption and area occupation because an additional circuit is required to capture the image data from the ISNS. We could not allot sufficient power and area to the ALR circuit to enable the retina chip to be integrated with over 1000 ISNS pixels. Consequently, we decided to design an ALR circuit, with 16 pixels, taking into account the image similarity, power consumption, and area consumption. In the next section, we present the ALR circuit to autonomously manage the integration time for high-contrast sensitivity.

**Figure 5:**
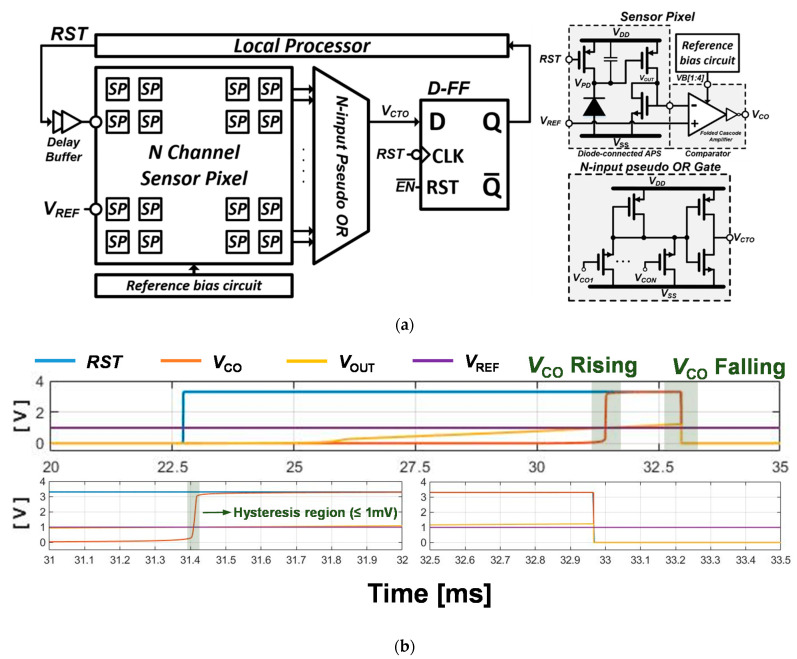
(**a**) Block diagram of the ambient light rejection circuit; (**b**) Simulation results of the sensor pixel.

Figure 5a shows a block diagram of the ALR circuit comprising an N-channel sensor pixel (SP), N-input pseudo OR gate, and D-flop flop sequential buffer. We composed 16-pixel SPs with the same structure as the ISNS pixel to compare the results. The SP consists of an APS, a common-source amplifier, and a comparator. We implemented an nMOS-input folded cascode amplifier for the comparator and a common reference bias generator to supply bias voltages to the comparator. *V_REF_*, the saturation voltage, is externally controlled to consider the variation of *I_max_* in Equation (2), caused by an impedance variation of the output stage. The image data through the SP are gathered on the N-input pseudo OR gate to inform the pixel saturation. We employed a pseudo OR gate, instead of a conventional logic gate, to efficiently process multichannel input and reduce power and area occupation. Therefore, we realized the indicator presented in Figure 3 using the SP and pseudo OR gates. In the algorithm, shown in Figure 3a, if the indicator detects pixel saturation, it decreases the integration time of the next sequence, and conversely increases the integration time of the next sequence if the pixel saturation is not detected. If the integration time is adjusted with the above mechanism when the pixel operates in a bright environment, a small integration time is applied to have SDR at high illuminance. On the contrary, the SDR at low illuminance is applied when operating in a dark environment. The correlation between integration time and SDR is shown in Figure 1c, which has the inverse relationship as described in Equation (2). In Figure 5, the output of the d-flipflop acts as an indicator and the local processor controls the integration time. 

Figure 5b shows the simulation results of the ALR circuit with a single SP. During the integration time, the *V_OUT_* gradually increases proportionally to the photocurrent, such as the ISNS pixel in Figure 1a. When the *V_OUT_* voltage is higher than the *V_REF_*, the *V_CO_* rises to logic “1” state, so SP is saturated. A *V_CTO_* via the pseudo OR gate indicates that there is one or more increased *V_CO_* in the SP array. The results of *V_CTO_* are stored at the end of the integration time and utilized for the ALR interpolation. At each end of the integration time, the *V_CTO_* is refreshed and delivered to a local processor. The stored *V_CTO_* is used to process the ALR interpolation, through a local processor, using the same procedure as in 

Figure 3a. For instance, based on the simulation result, the *V_CO_* shows logic ‘1′ at the end of the integration time. It means that the integration time on next sequence will be shorter than this sequence. More detail results about the ALR circuit will be presented on next section. 

## 3. Results

**Figure 6:**
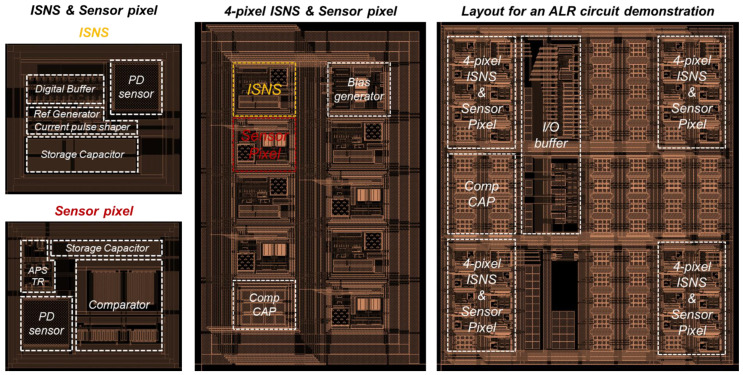
A layout of the proposed ALR circuit.

Figure 6 shows the micrograph of the proposed ALR circuit, where we integrated a 16-pixel ISNS and SP, as described. Each 4-pixel ISNS and SP are comprised of the ISNS, SP, bias generator, and compensation capacitor. We composed each 4-pixel ISNS and SPs separately to illuminate different light intensities. In the experiment, the ALR chip was tested to determine the *V_OUT_* difference between neighboring SPs. 

**Figure 7:**
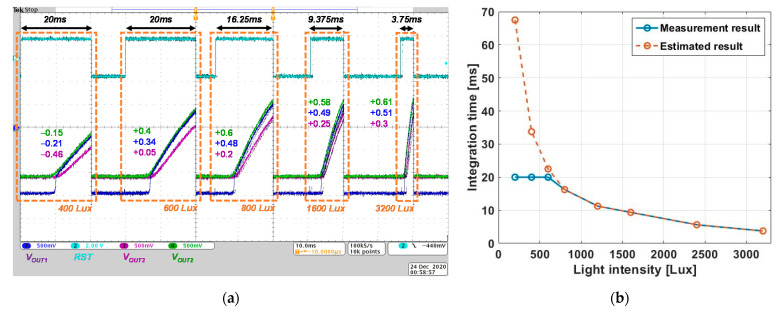
(**a**) The measured result versus a transient time and (**b**) integration time versus light intensity of the ALR circuit.

Figure 7 presents the measured results of the ALR circuit for each light intensity. We used custom-made LED light sources and a commercialized LED light source (66088-LED, Newport, Irvine, CA, USA) to project uniform light to the ALR circuit. Continuous light was irradiated to prevent distortion of the SP from a line scan camera [26,27] and the incident light intensity was measured using a commercialized illuminometer (TES 1336A, TES Corp., Taipe, Taiwan). The local processor was implemented with an FPGA board (Basys 3 Artix-7, Digilent Inc., Pullan, WA, USA).

Figure 7a displays the captured oscilloscopic images with an increase in the light intensity. *V_OUT,1-3_* show the time-dependent increments to incident light during the integration time. The integration time was inversely proportional to the incident light after *V_OUT_* was higher than saturation voltage, *V_REF_* as 2.2 V. Figure 7b shows the integration time for each light intensity. The ALR interpolation occurs after the time-to-saturation is inversely proportional to the light intensity, as described in Equation (2). The increment of the incident light intensity reduces the integration time, corresponding to the time that *V_OUT_* exceeds *V_REF_* (*V_OUT2_* in this study). From the measured results as shown in Figure 7a, it can be observed that the integration time is reduced to 16.25 ms to have an SDR near 800 lux where pixel saturation is detected. When operating at higher illuminance, the integration time keeps getting shorter so data are obtained from SDR at each illuminance. However, if *V_OUT_* does not reach *V_REF_*, the integration time increases until *V_OUT_* reaches *V_REF_*. We set the modulated integration time as 10 ms divided into four bits (0.625 ms as *T_LSB_*). Therefore, the maximum integration time is 20 ms with *T_REF_* as 10 ms. After the maximum integration time is achieved, the integration time cannot be longer even if the entire pixel is not saturated. In Figure 7b, the measured result shows that the integration time cannot be longer when the ALR circuit is exposed to 600 lux. The estimated result shows the required integration time for under 600 lux to induce *V_OUT_* saturation. The solution which makes the ALR circuit operate in dim condition will be described in the next section. As shown in Figure 7a, the modulated integration time followed the time-to-saturation of *V_OUT2_*. Accordingly, the middle point of the SDR was also changed corresponding to the incident light. Assuming that the SDR is proportional to the integration time in Equation (2), the middle of SDR is determined between 560 lux to 18200 lux, converted as 30 dB. It means that the ALR circuit offers additional 30 dB sensing dynamic range. Considering the SDR in Figure 1a is about 10 dB, the ALR circuit offers significant benefits, which makes it possible to sense high dynamic range on the ISNS pixel. 

Table 1 presents the power and area consumption of the ALR circuit. From the results of the simulation and the layout, we estimated that the power and area consumption of the ALR circuit comprised 16-SP, the pseudo OR gate, and the bias generator. The power consumption and area occupation are dominated by the SP owing to the presence of not just the 16-pixel SP on the ALR circuit. In the ALR circuit, shown in Figure 5a, the SP was constructed with the folded cascode amplifier to achieve sufficient gain of the comparator. If we change the folded cascode amplifier as the self-bias amplifier [28,29] and design the SP without the diode-connected APS (by directly connecting the comparator with the ISNS), we can compromise the area and power consumption. In the next section, we present the summary of the ALR circuit, comparison with relevant research, and future work. 

## 4. Conclusions and Discussion

In this paper, we present a novel ALR circuit that autonomously enhances contrast sensitivity to provide convenience and assistance to blind people suffering from retinal diseases such as retinitis pigmentosa and age-related macular degeneration. First, we introduced the ALR algorithm and verified the efficacy of the algorithm in through MATLAB simulations. Here, the main constraints are power consumption, area occupation, and interpolation efficacy. By optimizing the trade-off between these constraints, we designed an ALR circuit with 16 pixels. Although the 16 pixels are only 0.3% of all pixels, the image similarity is over 90%, and the interpolated image is visually similar to the original image. This is an optimization procedure for the ALR interpolation, which provides a guideline to determine the trade-off between the interpolation efficacy, and power and area occupation. In addition, by performing the optimization procedure, we could reduce the power consumption and area occupation compared to the conventional image-processing unit.

In the experimental results, shown in Figure 1, we used a 3-kΩ resistor as the electrode impedance. However, a tissue-electrode interference (ETI), modeled as an electrode impedance, should be changed with the size, geometry, and material of the electrode [30,31,32]. To compensate for the variation from the electrode, we decided to design an ALR circuit with adjustable *V_REF_*. As mentioned previously, the purpose of the ALR circuit is to keep the ISNS pixel operating in the SDR by preventing pixel saturation. Thus, if the *V_REF_* is to be lower than that obtained when the ISNS pixel generates the stimulation current, under *I_max_*, the ALR circuit autonomously makes the entire ISNS pixels continuously operate, without pixel saturation. The proposed ALR circuit was implemented on a silicon chip using a DongBu Hi-tek 0.35 μm CMOS process, which occupies an active area of ~190 µm^2^. When 16 reference pixels to reject the ambient light operate, it dissipates of 3.3 mW that is low enough to work with high-density stimulation pixels on a single chip. In addition, a maximum ALR feedback response time of 1.6 s was measured at a light intensity of 20,000 lux that in the worst case destroys a human retina. Therefore, a response time less than 1.6 s will be fine for the blind who implant the retinal chip to move around in their daily life.

The ALR algorithm implemented by the ASIC is experimented in a customized test bench. As shown in Figure 7a, different light intensities were irradiated on each pixel, and the results of this are clearly shown in Figure 7b. Theoretically, the ALR circuit offers the additional sensing dynamics for the ISNS pixel and will be autonomously worked. As a result, the integration time is autonomously regulated depending on the incident light intensity. However, the integration time could not be sufficiently stretched to operate under dim condition, such as under 400 lux. This implies that the ALR circuit provides a significant assistance to view an image under bright conditions, while it is ineffective under dim conditions. Two possible solutions can be considered: decreasing the accumulation capacitor and increasing the reference integration time *T_REF_*. Even though these have advantages as well as disadvantages, we will use both solutions, which is helpful in designing high-density retinal implants. Consequently, the proposed ALR circuit autonomously adapts the ISNS pixel to the incident light. In addition, we present the simulation results to ensure the ALR interpolation efficacy, considering the power and area consumption.

Electrical performance of the proposed work is summarized on Table 2 along with other previous works for comparison. The prior literature presented in [8,9,21,33] only has stimulation pixels. Although Park et al. [7] developed an edge stimulation method to increase contrast sensitivity, it cannot compensate for pixel saturation caused by the ambient light. Rothermel et al. [34,35] proposed an ambient light rejection technique that operates with 3025 stimulation pixels. The scheme shows a possibility that the ambient-light compensation can be applied for high-density stimulation pixels more than 3000. However, it requires 100 reference pixels to measure a saturation status, which can induce high-power consumption and a large area on the silicon chip. Our compensation technique proposed in this work requires few reference pixels due to the similarity optimization as shown in Figure 4b. According to the simulation result presented previously, 16 reference pixels among 64 × 64 stimulation pixels are enough to cancel out the deleterious effect of the ambient light. Therefore, our compensation work, which requires a pixel loss rate of 0.4% (=16 reference pixels/4096 stimulation pixels), is more efficient than the previous research [35] that demands the rate of 3.3% (=100 reference pixels/3025 stimulation pixels). In future work, we will design a retina implant integrated with over 2000 ISNSs on a single chip and will apply this refined chip for clinical trial.

## Figures and Tables

**Table 1:** The power consumption and area occupation for the ALR circuit.

	Power Consumption (μW)	Area Occupation (μm^2^)
ALR circuit	1748.88 (100%)	189,744.9 (100%)
Sensor pixel	93.68 (85.71%)	97 × 114 (93.24%)
ISNS pixel	56.4	97 × 114
16:1 OR gate	-	42.4 × 38.21 (0.85%)
Reference generator	250 (14.77%)	156.6 × 71.5 (5.9%)

* The period and duty cycle of the integration time for the ISNS and SP pixels are 50 ms and 40%, respectively, when considering the flicker-free vision. This result was calculated without power consumption on a simulated current.

**Table 2:** Specification comparison of retina prosthesis ASICs.

	TBioCAS’20 [7]	TED’20 [9]	TBioCAS’14 [21]	EMBC’20 [34]	Ophthalmol’20 [8,33]	This Work
Technology	0.18 μm	Custom	0.35 μm BCD	0.18 μm HV	Custom	0.35 μm
Supply power	Wireless coil	Wireless coil	Wireless coil	Wireless coil	Photovoltaic	Wireless coil
Electrode location	Sub-retina	Sub-retina	Sub-retina	Sub-retina	Sub-retina	Sub-retina
Stimulus approach	Simultaneous	Simultaneous	Sequential	Sequential	Simultaneous	Sequential
Pixel number	1225	100	128	3025	378	256
Pixel size (μm^2^)	84.3 × 86.6	400 × 400	50 × 55	51.5 × 51.7	7500	97 × 114
Chip size (mm^2^)	5 × 3.45	4 × 4	2.5 × 1.2	3.14 × 3.94	2 × 2	5 × 4
Stimulus current [loading parameter]	≤3 mA (10 kΩ resistor)	≤3 μA (PBS solution)	≤300 μA (10 kΩ resistor)	≤18 μA (PBS solution)	N/A	≤150 μA (10 kΩ resistor)
Supply voltage	± 1.6 V	5 V	12 V	± 1.6 V	N/A	±1.6 V
Application	- Edge only stimulation - Temperature sensor	N/A	N/A	- Ambient light rejection - High-pass filter	N/A	- Ambient light rejection
Power consumption	2.7 mW	320 μW	N/A	N/A	N/A	3.2 mW

* Power consumption is calculated excluding stimulation current.

## Data Availability

The data presented in this study are included in this article.
